# Supplementary material for: Inhibitors of the Actin-Bundling Protein Fascin-1 Developed for Tumor Therapy Attenuate the T-Cell Stimulatory Properties of Dendritic Cells
Source: Cancers (Basel). 2022 May 31;14(11):2738. doi: 10.3390/cancers14112738 (PMC9179534; doi:10.3390/cancers14112738)
Supplement: Supplementary file 1 [file cancers-14-02738-s001.zip › cancers-1742514-supplementary.pdf]

Supplementary Material

Inhibitors of the Actin-Bundling Protein Fascin-1 Developed for Tumor Therapy Attenuate the T-Cell Stimulatory Properties of Dendritic Cells

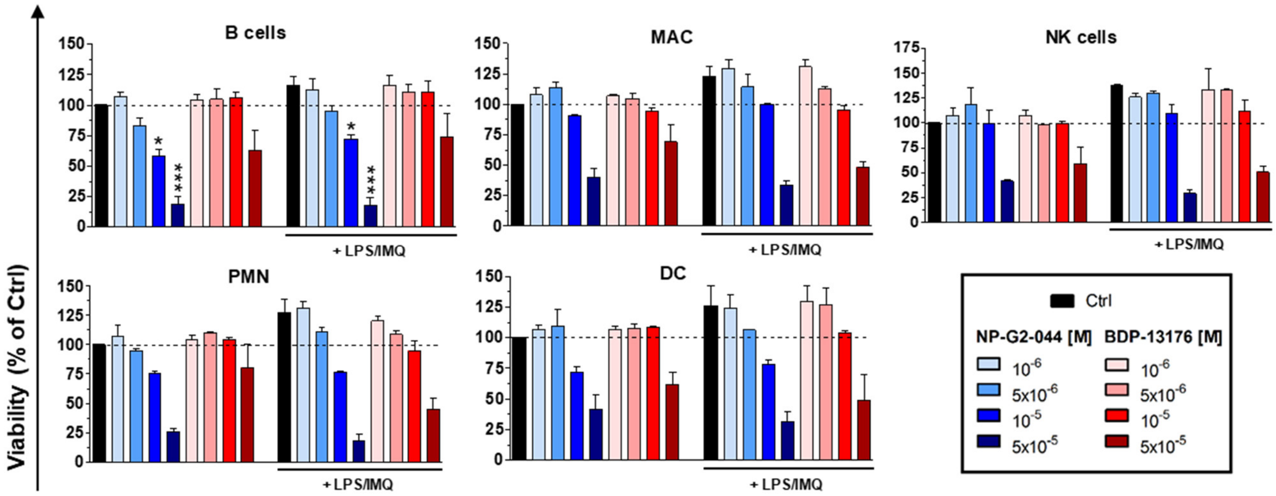

Figure S1. NP-G2-044 at high concentration attenuates the viability of splenic B cells. Spleen cells were incubated over night with Fscn1 inhibitors and stimulated as described (see Figure 1). Splenic immune cell populations were delineated by sequental gating as described [1]. Viabiliy of splenic immune cell populations was assessed by flow cytometric analysis. Graphs denote the frequency of FVD-negative viable cells (mean±SEM of 2–6 experiments), normalized to the Ctrl. Graphs denote MFI (mean±SEM of 6 experiments) given relative to expression by the control (Ctrl) group. Statistical differences versus * Ctrl are indicated (one way ANOVA, Tukey test). * $p < 0.05$, *** $p < 0.001$.

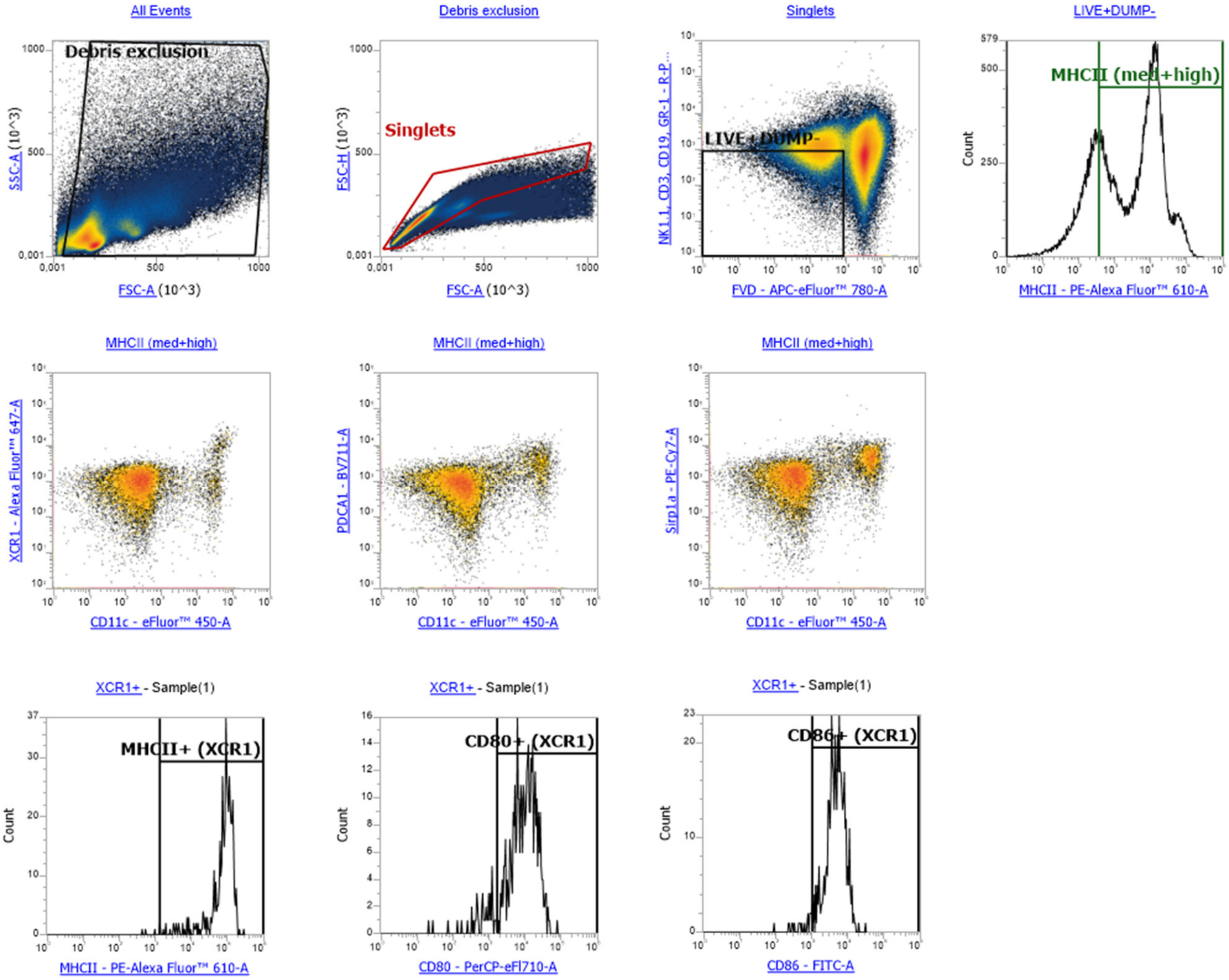

Figure S2. Gating strategy of splenic DC populations. After exclusion of debris, doublets, dead cells and non-DC (CD19⁺ B cells, CD3⁺ T cells, NK1.1⁺ NK cells and GR-1⁺ myeloid cell types gated out [`dump channel]), splenic DC populations were identified according to coexpression of CD11c and XCR1 (cDC1), CD172/SIRPa (cDC2) and CD317/PDCA1 (pDC). For each DC population, expression intensities of MHCII, CD80 and CD86 were tested.

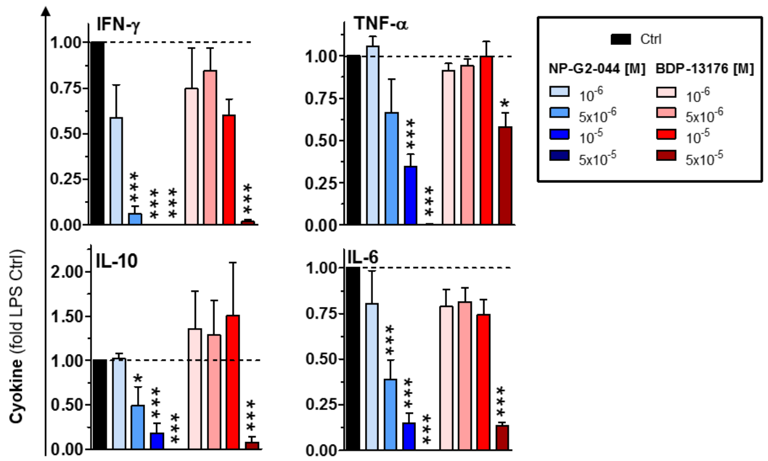

Figure S3. NP-G2-044 affects cytokine production of stimulated spleen cells. Spleen cells were incubated over night with NP-G2-044 and stimulated as described (see Figure 1). Cytokine concentrations in culture supernatants were determined by CBA (mean ± SEM of 3–4 experiments). Statistical differences versus * Ctrl are indicated (one way ANOVA, Tukey test). * $p < 0.05$, *** $p < 0.001$.

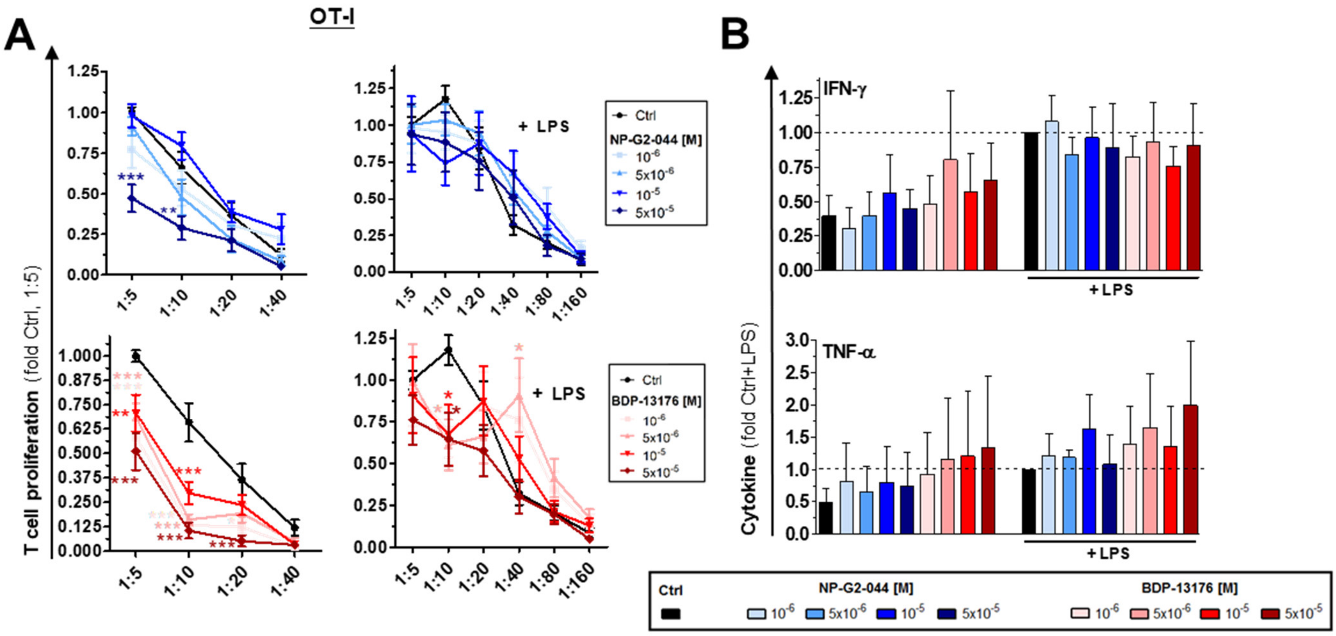

Figure S4. BMDC pretreated with Fscn1 inhibitors confer lower CD8+ T cell stimulatory capacity. (**A**,**B**) BMDC were treated, incubated and co-cultured as described (see Figure 4) with CD8+ (OT-I) T cells. (**A**) Proliferation of CD8+ T cells was assessed by incorporation of 3H-thymidin applied for the last 16 h of 3-4 days of coculture. Data denote the mean±SEM of 3–4 compiled experiments performed in triplicates and given relative to the according Ctrl condition (1:5). (**B**) Prior to application of ^{3}H-thymidine aliquots of DC/T cell coculture supernatants (1:5) were retrieved and cytokine contents in CD8+ cocultures were assayed by CBA. Data denote the mean±SEM of 3-4 experiments given relative to Ctrl+LPS. (**A**) Statistical differences versus *Ctrl are indicated (one way ANOVA, Tukey test). * $p < 0.05$, ** $p < 0.01$, *** $p < 0.001$.

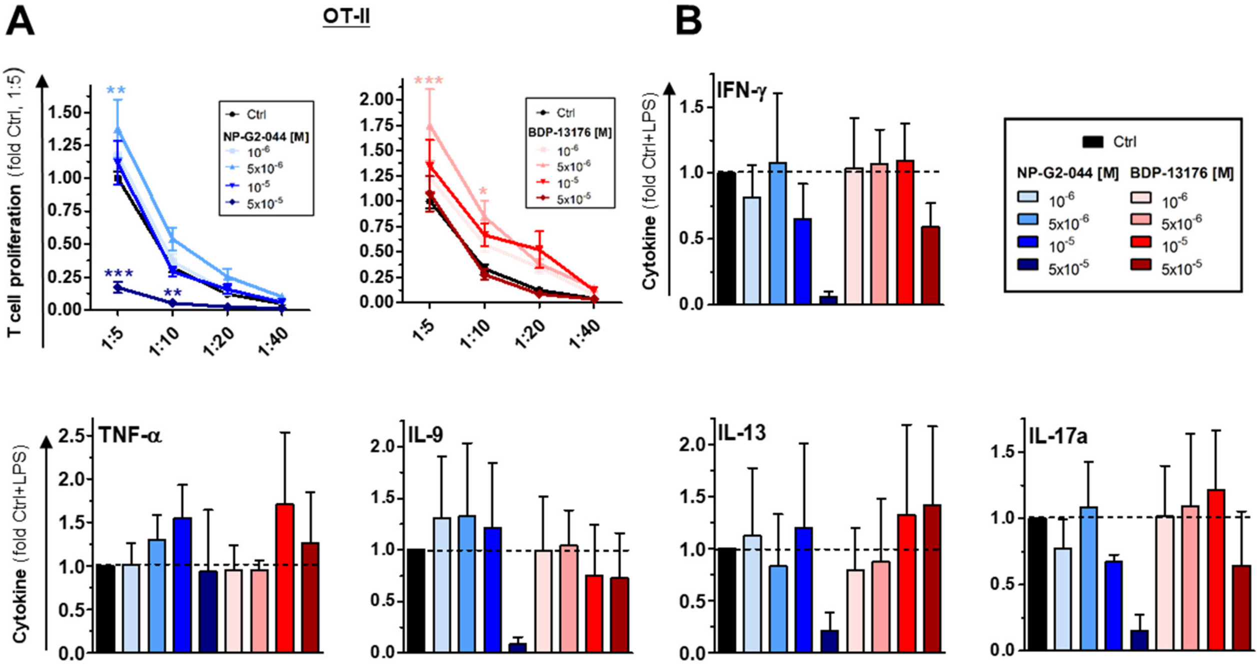

Figure S5. BMDC pretreated with Fscn1 inhibitors confer lower CD4+ T cell stimulatory capacity. (**A**,**B**) BMDC were treated without final LPS stimulation and co-cultured as described (see Figure 4) with CD4+ (OT-II) T cells. (**A**) Proliferation of CD4+ T cells was assessed by incorporation of 3H-

thymidin applied for the last 16 h of 3–4 days of coculture. Data denote the mean±SEM of 2-3 compiled experiments performed in triplicates and given relative to the according Ctrl condition (1:5). (**B**) Prior to application of 3H-thymidine aliquots of DC/T cell coculture supernatants (1:5) were retrieved and cytokine contents in CD4⁺ cocultures were assayed by CBA. Data denote the mean±SEM of 4–6 experiments given relative to Ctrl. (**A**) Statistical differences versus * Ctrl are indicated (one way ANOVA, Tukey test). ** $p < 0.01$, *** $p < 0.001$.

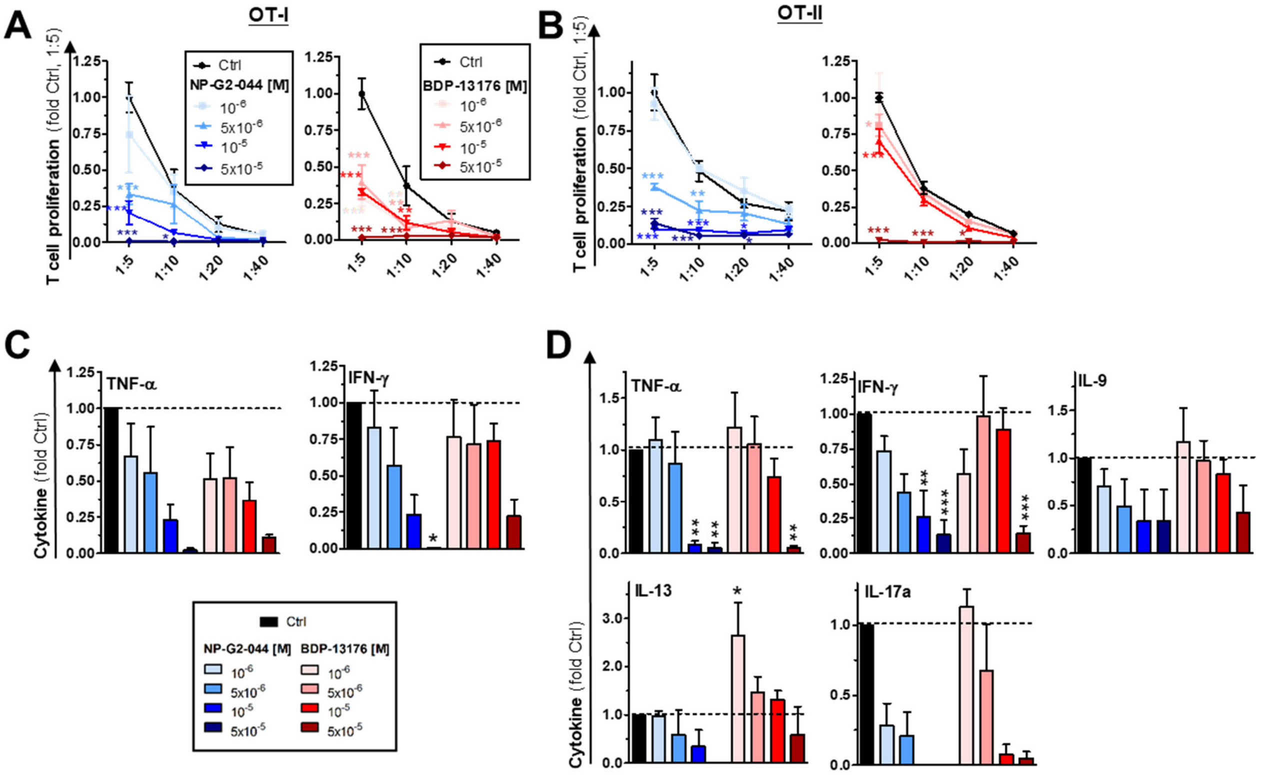

Figure S6. Application of Fscn1 inhibitors during DC/T cell coculture results in impaired T cell proliferation. (**A-D**) BMDC were incubated with OVA protein (5 µg/mL) and treated 3 h later with Fscn1 inhibitors as indicated. On the next day, samples were harvested, washed and serially titrated numbers of DC were cocultured with immunomagnetically sorted OVA peptide-specific CD8⁺ (OT-I) (**A, B**) and CD4⁺ (OT-II) (**C, D**) T cells (each 5 × 10^4/100 µl) in triplicates in 96 well plates. (**A**, **C**) Proliferation of CD8+ (**A**) and CD4⁺ (**C**) T cells was assessed by incorporation of ^{3}H-thymidin applied for the last 16 h of 3–4 days of coculture. Data denote the mean±SEM of 2 compiled experiments performed in triplicate and given relative to the according Ctrl condition (1:5). (**B**, **D**) Prior to application of ^{3}H-thymidine aliquots of DC/T cell coculture supernatants (1:5) were retrieved and cytokine contents in (**B**) CD8⁺ and (**D**) CD4⁺ cocultures were assayed by CBA. Data denote the (mean±SEM of 3–5 experiments given relative to Ctrl. (**A-D**) Statistical differences versus * Ctrl are indicated (one way ANOVA, Tukey test). * $p < 0.05$, ** $p < 0.01$, *** $p < 0.001$.

References

1. Bednarczyk, M.; Medina-Montano, C.; Fittler, F.J.; Stege, H.; Roskamp, M.; Kuske, M.; Langer, C.; Vahldieck, M.; Montermann, E.; Tubbe, I.; et al. Complement-Opsonized Nano-Carriers Are Bound by Dendritic Cells (DC) via Complement Receptor (CR)3, and by B Cell Subpopulations via CR-1/2, and Affect the Activation of DC and B-1 Cells. *Int. J. Mol. Sci.* **2021**, *22*, 2869. https://doi.org/10.3390/ijms22062869.
